# Transcriptome Analysis of miRNAs Involved in the Myogenic Differentiation of Goat Skeletal Muscle Satellite Cells

**DOI:** 10.3390/cells15060519

**Published:** 2026-03-13

**Authors:** Runxiao Luo, Tao Zhong, Linjie Wang, Shizhong Yang, Li Li, Hongping Zhang, Siyuan Zhan

**Affiliations:** 1 Farm Animal Germplasm Resources and Biotech Breeding Key Laboratory of Sichuan Province, College of Animal Science and Technology, Sichuan Agricultural University, Chengdu 611130, China; luoruxiao@stu.sicau.edu.cn (R.L.); zhongtao@sicau.edu.cn (T.Z.); wanglinjie@sicau.edu.cn (L.W.); lily@sicau.edu.cn (L.L.); zhp@sicau.edu.cn (H.Z.); 2 Liangshan Academy of Agricultural Sciences, Xichang 615000, China; y111sz@163.com

**Keywords:** myogenic differentiation, goats, MicroRNAs, skeletal muscle satellite cell, transcriptome

## Abstract

Skeletal muscle myogenesis is a crucial factor influencing meat production in livestock. MicroRNAs (miRNAs) play a significant role in skeletal muscle myogenesis. The objective of this study was to identify key miRNAs involved in the process of goat skeletal muscle satellite cell (MuSC) differentiation into myotubes. We performed miRNA expression profiling analysis during the proliferation phase (cultured in growth medium, GM) and the differentiation phase (cultured in differentiation medium for 1 day and 5 days, classified as DM1 and DM5, respectively) of goat skeletal muscle satellite cells (MuSCs). A total of 1846 miRNAs were identified in MuSC samples, of which 677 differentially expressed miRNAs (DEmiRNAs) were screened through pairwise comparisons across three groups (GM vs. DM1, GM vs. DM5, and DM1 vs. DM5), and the results were further confirmed by a quantitative real-time PCR assay. Time-series expression profiling facilitated the categorization of the DEmiRNAs into eight distinct clusters, one of which demonstrated a significantly downregulated expression pattern (*p* < 0.05). Functional enrichment analysis revealed that the target genes of DEmiRNAs are involved in several pathways that are critical for myogenesis, including Hippo, TGF-β, MAPK and cell adhesion molecules. Interaction network analysis identified 19 miRNAs and 56 mRNAs associated with muscle cell development. Notably, novel-m0047-5p emerged as a key regulator, exhibiting strong negative correlations (r = −0.88 to −0.89, q < 0.01) with muscle-related target genes FOSB, CPT1B, and MYOZ2. These findings elucidate miRNA-mediated regulatory networks in goat myogenesis and provide candidate molecular targets for genetic improvement of meat production traits.

## 1. Introduction

China’s goat industry has grown substantially over the past decade, mainly from increased meat production. Goat meat is popular among consumers looking for lean protein because it has a distinctive taste and less fat than other red meats [1]. In adult ruminants, skeletal muscle makes up 40–60% of body mass and strongly influences both production efficiency and meat quality [2]. Muscle development proceeds through several stages: satellite cells become activated, myoblasts multiply and differentiate, these cells merge into myofibers, and numerous genes are expressed along the way [3]. Today’s molecular breeding work calls for research into what happens at the cellular and molecular level during these stages, especially regarding myogenic differentiation.

Advances in high-throughput sequencing have led to the discovery of many noncoding RNAs, including miRNAs, lncRNAs, and circular RNAs, involved in muscle growth and development [4]. These small RNAs, usually 21–23 nucleotides in length, bind to 3′-untranslated regions of their target mRNAs via seed sequence complementarity, leading to transcript degradation or translational repression [5]. Evidence from multiple species demonstrates that miRNAs are critical regulators of myogenesis. For instance, Chen et al. [6] reported that miR-1 and miR-206 suppress Pax7 expression, which reduces satellite cell proliferation while enhancing myogenic differentiation. Similarly, miR-193b-3p induces its expression through binding to the 3′ untranslated region of IGF2BP1, thereby facilitating myogenic differentiation in goat myoblasts [7]. Ma et al. found that miR-205 regulates myoblast fusion by targeting the 3′ untranslated region of TMEM8c, a key fusogenic factor, thus suppressing its expression and consequently inhibiting porcine myoblast fusion [8]. In addition, miRNAs also regulate transitions between muscle fiber types. miR-196-5p regulates chicken myogenesis and promotes the development of slow-twitch myofibers through targeting PBX1 [9]. Research on the *longissimus dorsi* muscle of Rongchang pigs reveals that miR-152 promotes the development of slow-twitch muscle fibers by targeting and downregulating uncoupling protein-3, thereby modulating skeletal muscle fiber composition [10]. Thus, characterizing miRNA expression and their regulatory mechanisms during goat myogenic differentiation may elucidate the molecular processes governing muscle development, thereby offering a theoretical foundation for the genetic improvement of meat production performance and meat quality traits in meat-type goats.

In this study, RNA sequencing was employed to identify differentially expressed miRNAs during the differentiation of goat skeletal muscle satellite cells, and the observed differences in expression levels were subsequently validated through quantitative real-time PCR (qRT-PCR) assays. To delineate the dynamic regulatory events underlying myogenic differentiation, we conducted a time-series expression analysis at three key stages: GM, DM1, and DM5. Through functional analysis and the construction of miRNA-mRNA interaction networks, we identified candidate miRNAs potentially involved in goat skeletal myogenesis. These findings provide molecular targets and a theoretical framework for understanding the regulatory mechanisms of goat myogenesis and for the genetic improvement of meat quality.

## 2. Materials and Methods

### 2.1. Preparation of Cell Cultures and Samples

One newborn Chengdu Ma goat was randomly selected from a contemporaneous birth cohort using computer-generated random numbers for primary skeletal muscle stem cell isolation from the *longissimus dorsi* [11]. Briefly, following humane euthanasia, LD tissue was harvested, rinsed with sterile phosphate-buffered saline (PBS; Hyclone, Logan, UT, USA), and trimmed to remove visible connective tissue. The tissue was minced and digested with 0.2% (*w*/*v*) Pronase (Sigma-Aldrich, St. Louis, MO, USA) at 37 °C for 1 h, followed by centrifugation at 1500× *g* for 6 min. The pellet was washed twice with PBS, resuspended in growth medium [DMEM high glucose (Gibco, Thermo Fisher Scientific, Waltham, MA, USA) supplemented with 15% fetal bovine serum (FBS; Gibco, Thermo Fisher Scientific, Waltham, MA, USA) and 2% penicillin/streptomycin (P/S; Hyclone, Logan, UT, USA)], and filtered through a 70-μm cell strainer (BD Biosciences, Franklin Lakes, NJ, USA). The filtrate was centrifuged at 800× *g* for 5 min to obtain the crude MuSC fraction. Subsequently, cells were purified by discontinuous Percoll density gradient centrifugation (20%, 40%, and 90%, from top to bottom; Sigma-Aldrich, St. Louis, MO, USA) at 1800× *g* for 50 min. The MuSC-enriched fraction at the 40%/90% interface was collected. Purified MuSCs were characterized by Pax7 immunostaining (rabbit anti-Pax7 antibody, 1:100; Boster, Biological Technology, Wuhan, China) and used for subsequent experiments. MuSCs were seeded at approximately 2 × 104 cells per well in 6-well plates and cultured in growth medium (GM) containing DMEM base (Catalog No. C11995500BT) with 10% FBS and 2% penicillin/streptomycin (Solarbio, Beijing, China). Upon reaching 80–90% confluency, cells were transferred to differentiation medium (DM) with the same DMEM base supplemented with 2% horse serum and 2% penicillin/streptomycin to induce myogenic differentiation. Cells were maintained at 37 °C in a 5% CO_2_ atmosphere, with medium replacement every 48 h. Proliferative MuSC samples were labeled GM, whereas cells subjected to 1-day or 5-day differentiation were termed DM1 and DM5, respectively. Three biological replicates were prepared for each condition and preserved at −80 °C until RNA isolation. After removing the culture medium, cells were immediately lysed in 1 mL of Trizol Reagent (Invitrogen, Thermo Fisher Scientific, Waltham, MA, USA) per well. The lysates were stored at −80 °C for up to three months until RNA extraction.

### 2.2. Total RNA Extraction and Library Construction

Total RNA was extracted from the Trizol lysates following the provided protocol. RNAs ranging from 18 to 30 nucleotides in length were isolated using polyacrylamide gel electrophoresis (PAGE). Following 3′ adapter ligation, products of 36–44 nucleotides were subsequently isolated. After ligation of 5′ adapters, the products were reverse-transcribed and PCR-amplified. PCR amplicons ranging from 140 to 160 bp were isolated for cDNA library construction and subsequently sequenced using the Illumina NovaSeq X Plus system (Illumina, San Diego, CA, USA).

### 2.3. Small RNA Sequencing Data Processing and Analysis

Quality control was applied to raw reads based on these criteria: (1) sequences with >1 base having Q ≤ 20 or ambiguous nucleotides (N) were discarded; (2) sequences lacking 3′ adapters were filtered out; (3) those bearing 5′ adapters were rejected; (4) sequences possessing both 3′ and 5′ adapters without an insert were discarded; (5) sequences containing poly(A) tails within the insert region were removed; and (6) fragments <18 nt (adapter-inclusive) were eliminated. Quality-filtered tags were mapped against small RNA databases (GenBank Release 209.0 and Rfam) and the goat reference genome to exclude non-miRNA sequences such as exonic, intronic, and repetitive elements. Filtered clean tags were aligned to miRBase using Bowtie (version 1.1.2; Johns Hopkins University, Baltimore, MD, USA) for known miRNA identification, and miRDeep2 (Max Delbrück Center, Berlin, Germany) was applied to predict novel miRNAs. Expression values were normalized as transcripts per million (TPM) according to the equation: TPM = (miRNA read count × 10^6^)/total clean tags. Principal component analysis (PCA) and expression heatmaps were produced in R (http://www.r-project.org/).

### 2.4. Identification and Analysis of Differentially Expressed miRNAs

Differentially expressed miRNAs across pairwise comparisons were assessed using the edgeR package (Bioconductor, Fred Hutchinson Cancer Center, Seattle, WA, USA). Significant DEmiRNAs were identified as those miRNAs showing a fold change of at least two and a *p*-value below 0.05. Expression values for all miRNAs were converted to transcripts per million (TPM) format using the calculation: TPM = (miRNA read count/total clean tag count) × 10^6^. Sample relationships and expression patterns were visualized through principal component analysis (PCA) and hierarchical clustering heatmaps using R software (version 4.1.1; R Foundation for Statistical Computing, Vienna, Austria).

### 2.5. STEM Trend Analysis and Functional Enrichment

To analyze the temporal expression patterns of differentially expressed microRNAs (DEmiRNAs) during differentiation, trend analysis was conducted using Short Time-series Expression Miner (STEM; Carnegie Mellon University, Pittsburgh, PA, USA). Gene expression data were normalized to the proliferation stage, with samples ordered chronologically (GM, DM1, DM5) and transformed to log2 relative expression values. STEM parameters were set with a maximum output of 20 model profiles and a unit change threshold of 1.0 between adjacent time points, corresponding to a minimum twofold expression change. This threshold ensured the capture of genes with significant expression variation, grouping transcripts with analogous temporal trends into coherent profiles. Clusters with a *p*-value ≤ 0.05 were deemed statistically significant and subsequently underwent Gene Ontology (GO) functional enrichment and Kyoto Encyclopedia of Genes and Genomes (KEGG) pathway analyses.

### 2.6. Prediction of DEmiRNA Targets and Functional Annotation

DEmiRNA targets were identified with Miranda (v3.3a; Memorial Sloan-Kettering Cancer Center, New York, NY, USA) and TargetScan (v7.0; Whitehead Institute, Cambridge, MA, USA). Genes predicted by both algorithms were retained as high-confidence targets. Using the hypergeometric test, GO enrichment analysis was carried out on the GO database. KEGG pathway analysis was performed via hypergeometric testing against the KEGG database. GO terms and KEGG pathways with *p* < 0.05 were deemed significantly enriched for DEmiRNA target genes.

### 2.7. Interaction Network Analysis

DEmiRNAs and their target genes consistently identified across all pairwise comparisons were filtered and intersected with differentially expressed mRNAs to explore potential miRNA–mRNA regulatory interactions. miRNA–mRNA interaction networks were constructed and visualized using Cytoscape v3.10.0 (National Resource for Network Biology, San Diego, CA, USA). The mRNA expression profiles were derived from the same batch of goat MuSC samples (GM, DM1, DM5; three biological replicates per group) as described in our previous study [12], ensuring consistent experimental conditions. To validate these predicted interactions, Pearson’s correlation analysis was performed between each DEmiRNA and its predicted target mRNA across all nine samples. miRNA–mRNA pairs with r < 0 and q < 0.05 (Benjamini–Hochberg correction) were considered significantly negatively correlated.

### 2.8. Verification and Statistical Analysis

Six DEmiRNAs were randomly selected to validate the miRNA sequencing data. miRNA primers were designed with MiRNA Design V1.01 (Vazyme, Nanjing, China; Table 1), and U6 served as the intern al control. First-strand cDNA was synthesized with the miRNA 1st Strand cDNA Synthesis Kit (stem-loop) (Vazyme, Nanjing, China; #MR101) and subsequently amplified using SYBR Premix Ex Taq™ II (Takara, Dalian, China). Each sample was analyzed with three technical replicates. Relative expression was calculated via the 2^−ΔΔCt^ method and expressed as mean ± SD. Data visualization was performed in GraphPad Prism 9 (GraphPad Software, San Diego, CA, USA). Expression trends (upregulation or downregulation) were qualitatively compared with RNA-seq data to confirm sequencing reliability.

## 3. Results

### 3.1. MuSC Differentiation Program

**Figure 1:**
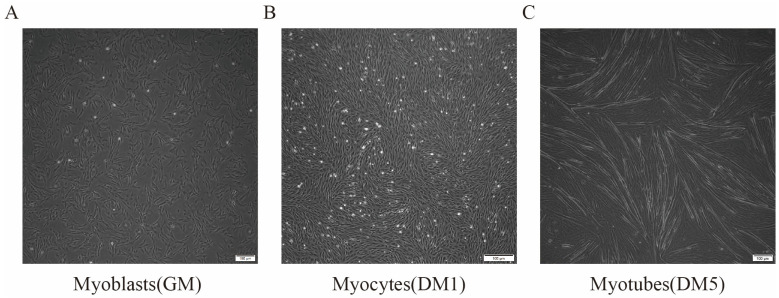
MuSC Differentiation Program. (**A**) MuSCs were cultured in growth medium until they reached 80% confluence, representing the myoblast stage. (**B**) MuSCs were then cultured in differentiation medium for one day, transitioning to the myocyte stage. (**C**) MuSCs were cultured in differentiation medium for five days, developing into myotubes.

Skeletal muscle satellite cells (MuSCs) in growth medium looked long and thin and divided quickly (GM samples; Figure 1A). When the cells covered most of the dish, they were moved to a solution that helps them turn into muscle (DM). After one day in this new solution, they became long muscle cells (DM1 samples; Figure 1B). By day five, these cells formed long tubes with many centers (DM5 samples; Figure 1C). Finally, samples from GM, DM1, and DM5 were collected to determine which genes were activated.

### 3.2. Summary of Small RNA Sequencing

**Figure 2:**
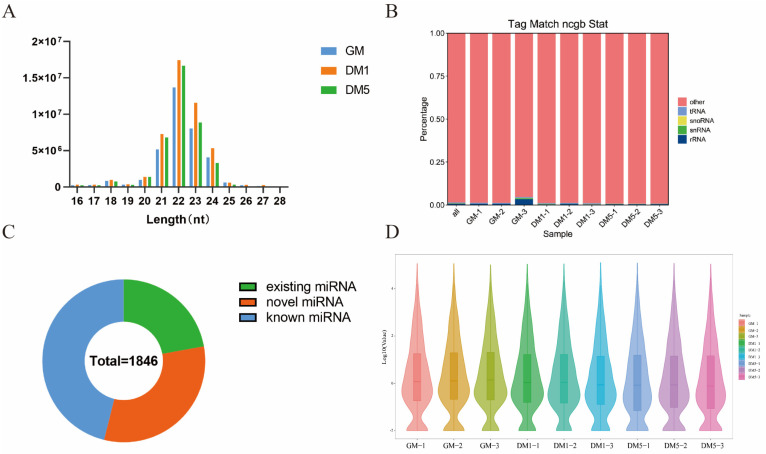
Overview of Small-RNA Sequencing. (**A**) The distribution of the lengths of clean reads in the MuSCs is presented. (**B**) Tag abundance statistics for genome alignment are provided. (**C**) The classification and identification of miRNAs are detailed. (**D**) A violin plot illustrates the expression levels of the samples.

In this study, nine samples yielded a total of 123,359,750 clean reads. Following quality control (removal of low-quality sequences, adapter contaminants, and poly(A)-containing reads), high-quality reads constituted ≥99.12% of total reads per replicate. In comparison, the proportion of clean tags was at least 99.14% (Table 2). Statistical analysis of the small RNA length distribution showed that 22-nucleotide (nt) reads were the most common (Figure 2A). Additionally, the percentage of high-quality reads with lengths between 21 nt and 23 nt ranged from 77.4% to 82.7% (Supplemental Table S1). Rfam database mapping showed that ~1% of tags corresponded to non-coding RNA species (rRNA, snRNA, snoRNA, and tRNA) (Figure 2B). The remaining 99% of tags were used for further analysis. We detected 1846 miRNAs, consisting of 409 pre-annotated miRNAs, 851 known miRNAs, and 586 novel miRNAs (Figure 2C). Next, the differences among samples were examined using density distribution plots of miRNAs and violin plots illustrating expression levels across the nine samples. Expression density and levels were consistent within each group, indicating reliable library construction, sequencing, alignment, and quantification. Conversely, samples from different groups showed distinct expression patterns, reflecting the miRNA profiles of skeletal MuSCs at various stages of proliferation and differentiation (Figure 2D).

### 3.3. Identification of Differentially Expressed miRNAs

**Figure 3:**
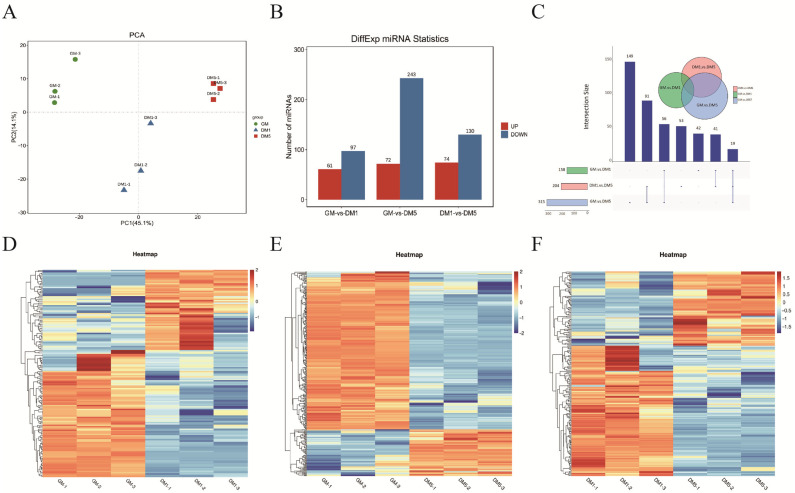
Identification of Differentially Expressed miRNAs. (**A**) Principal Component Analysis (PCA) of nine samples is conducted. (**B**) The number of up- and down-regulated miRNAs is reported. (**C**) An upset plot shows miRNA expression over time. (**D**–**F**) Hierarchical clustering analysis of differentially expressed miRNAs is performed through pairwise comparisons.

To explore miRNA expression profiles and investigate potential regulatory pathways during goat myoblast differentiation, we first performed principal component analysis (PCA) on sequencing data from 9 samples. The PCA results (Figure 3A) showed that the nine samples could be grouped into three distinct time points: GM, DM1, and DM5. Samples from each group clustered together, demonstrating high sequencing reliability. Differential miRNA expression across pairwise comparisons was assessed using the edgeR package, revealing 677 DEmiRNAs across all three comparisons: 158 (GM vs. DM1), 315 (GM vs. DM5), and 204 (DM1 vs. DM5) (Figure 3B; Supplemental Table S2). To identify key miRNAs involved in muscle development, an Upset plot analysis was performed on miRNAs from different stages. This analysis revealed 42, 149, and 53 stage-specific DEmiRNAs in the three comparisons, respectively, with 19 DEmiRNAs consistently expressed across all three stages (Figure 3C). Hierarchical clustering was performed to assess DEmiRNA expression profiles and reveal inter-sample variation. The results showed that samples from the same group formed strong clusters (Figure 3D–F).

### 3.4. Expression Trend and Enrichment Analysis

**Figure 4:**
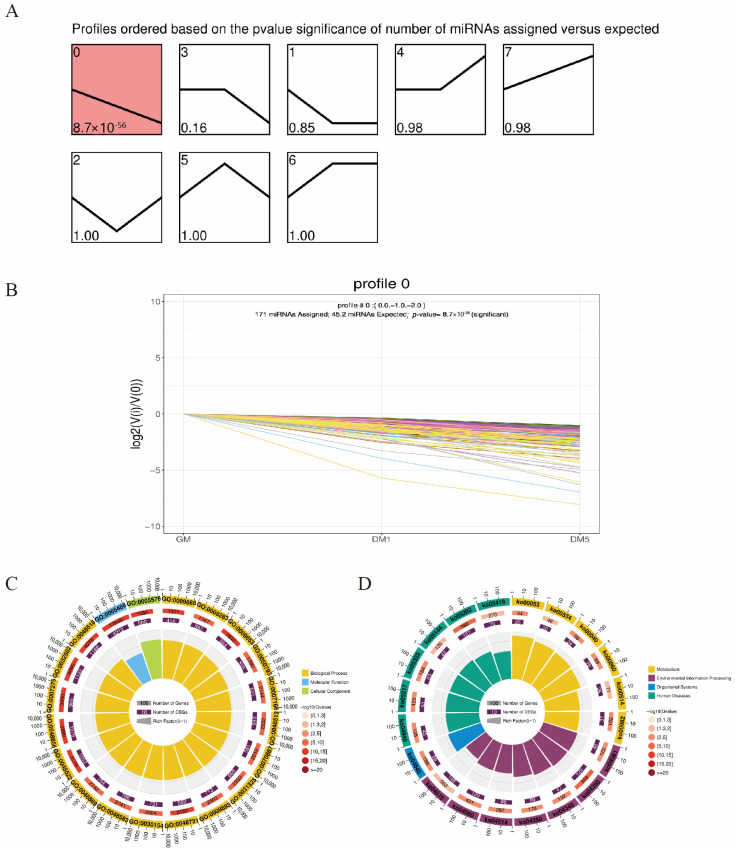
STEM and enrichment analysis of DEmiRNAs. (**A**) STEM clustering of DEmiRNA expression profiles (GM, DM1, DM5); red indicates significant clusters. (**B**) Expression dynamics of the 171 DEmiRNAs in profile 0 (continuous downregulation). (**C**) Top 20 GO terms enriched in target genes of profile 0 DEmiRNAs (**D**) Top 20 KEGG pathways enriched in target genes of profile 0 DEmiRNAs.

Cluster analysis of the expression patterns of DEmiRNAs across the GM, DM1, and DM5 stages identified a total of 451 DEmiRNAs. A total of eight temporal expression patterns were identified, among which profile 0 was significantly enriched and exhibited continuous downregulation (Figure 4A). Specifically, profile 0 comprised 171 DEmiRNAs (Figure 4B). We subsequently performed Gene Ontology (GO) and Kyoto Encyclopedia of Genes and Genomes (KEGG) enrichment analyses on their target genes. GO enrichment analysis revealed that these target genes were significantly enriched in biological processes related to development and proliferation, including developmental process (GO:0032502), cell differentiation (GO:0030154), cell proliferation (GO:0008283), and positive regulation of biological process (GO:0048518) (Figure 4C). Furthermore, KEGG enrichment analysis identified numerous pathways well known to be involved in myogenesis, such as the Hippo (ko04390), TGF-beta (ko04350), Hedgehog (ko04340), and MAPK (ko04010) signaling pathways (Figure 4D).

### 3.5. Prediction of miRNA Target Genes and Functional Enrichment Analysis

miRNAs regulate cellular processes by binding target mRNAs. We employed Miranda v3.3a and TargetScan v7.0 for target prediction. In the comparison between GM and DM1, 158 DEmiRNAs were found to target 7049 genes. Conversely, in the GM versus DM5 comparison, 315 DEmiRNAs targeted 7217 genes, while in the DM1 versus DM5 comparison, 204 DEmiRNAs targeted 7141 genes (Supplemental Table S3). We conducted GO and KEGG enrichment analyses on predicted DEmiRNA targets from each comparison to elucidate their functional roles. For the GM versus DM1 comparison, the target genes of DEmiRNAs showed significant enrichment in 686 GO terms (*p* < 0.05), including 622 terms related to biological processes (BPs), 56 to molecular functions (MFs), and 8 to cellular components (CCs) (Supplemental Table S4). The GM versus DM5 comparison showed enrichment of 623 GO terms (*p* < 0.05): 558 BP, 59 MF, and 6 CC terms (Supplemental Table S5). For DM1 versus DM5, 658 GO terms reached significance (*p* < 0.05), with 594 BP, 56 MF, and 8 CC terms (Supplemental Table S6).

**Figure 5:**
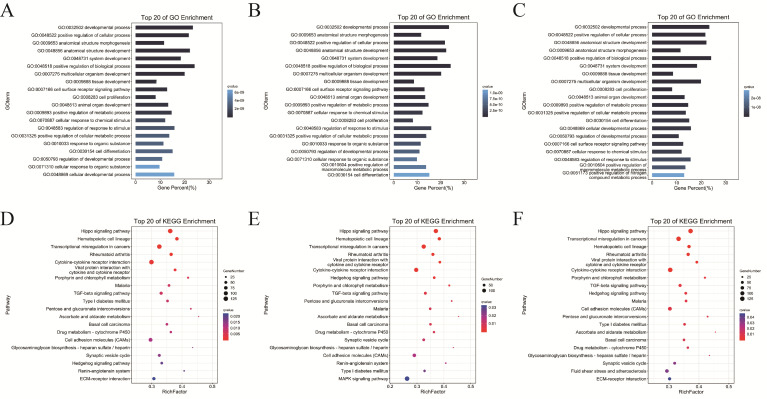
Target gene prediction and functional enrichment analysis of DEmiRNAs. (**A**) The top 20 most significant terms in biological processes in DM1 vs. DM5. (**B**) The top 20 significant terms of biological process in GM vs. DM1. (**C**) The top 20 significant terms of biological process in GM vs. DM5. (**D**) The top 20 significant pathways in DM1 vs. DM5. (**E**) The top 20 significant pathways in GM vs. DM1. (**F**) The top 20 significant pathways in GM vs. DM5.

The enrichment analysis results showed that the top 20 most significantly enriched biological processes across the three comparison groups are highly consistent, with 17 GO terms overlapping completely (Figure 5A–C). Notably, BPs related to muscle growth and development, such as morphogenesis and development of anatomical structures, tissue development, cell differentiation, and cell surface receptor signaling pathways, were consistently identified across all groups. Additionally, each comparison identified stage-specific enrichment terms, including cellular developmental processes prominent in the late differentiation stage.

The KEGG pathway enrichment analysis revealed significant enrichment of differentially expressed target genes in all three comparison groups, identifying 22, 23, and 22 enriched pathways, respectively (Supplemental Table S7). Pathways such as the Hippo signaling pathway, cytokine–cytokine receptor interaction, TGF-β signaling pathway, and cell adhesion molecules were consistently enriched across all groups, highlighting their essential roles in the regulatory mechanisms of skeletal muscle development (Figure 5D–F).

### 3.6. MiRNA-mRNA Interaction Network Analysis

**Figure 6:**
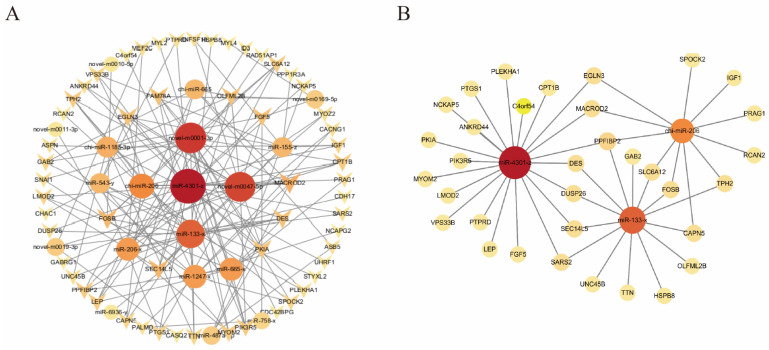
DEmiRNAs and DEmRNAs network analysis. (**A**) The DEmiRNAs and DEmRNAs interaction networks, including 19 DEmiRNAs and 56 DEmRNAs. (**B**) Interaction analysis of miR-4301-z, miR-133-x, and miR-206 with their DEmRNAs. Node color intensity indicates node degree (number of connections), with darker colors representing higher connectivity.

We identified overlapping DEmiRNAs and their corresponding target genes across the three comparative groups. These were then integrated with the mRNA differential expression profiles from our previous study [12]. Using Cytoscape, we built a regulatory network showing co-expression interactions between DEmiRNAs and DEmRNAs across various developmental stages of MuSCs. We identified 19 DEmiRNAs and 56 DEmRNAs with 149 interaction pairs (Supplemental Table S8). The most connected miRNAs were miR-4301-z (21 mRNA targets), novel-m0001-3p (17 targets), novel-m0047-5p (16 targets), miR-133-x (14 targets), and miR-206 (11 targets) (Figure 6A). miR-4301-z, for instance, targets DES, MYOM2, LMOD2, and LEP, genes that participate in myogenesis and muscle development. In contrast, miR-133-x and miR-206 are critical myogenic regulators that control genes associated with goat muscle differentiation (Figure 6B). Our results indicate that DEmiRNAs likely modulate myogenesis through transcriptional regulation, thereby influencing muscle formation. To validate these predicted interactions, Pearson correlation analyses were performed between the expression levels of the 19 DEmiRNAs and their 56 target DEmRNAs across all nine samples. Given that miRNAs typically negatively regulate their target mRNAs, we focused on inverse correlations with q < 0.05 (Benjamini–Hochberg correction). Of the 149 predicted pairs, 55 (37%) exhibited significant negative correlations (Supplemental Table S9), supporting the potential regulatory roles of these miRNAs. The strongest inverse correlations included chi-miR-665–GAB2 (r = −0.983, q < 0.001), novel-m0001-3p–CHAC1 (r = −0.962, q < 0.001), and novel-m0047-5p–MACROD2 (r = −0.951, q < 0.001). Notably, novel-m0047-5p showed significant negative correlations with 12 of its 16 predicted target mRNAs (75%), implying a particularly prominent regulatory role during myogenic differentiation.

### 3.7. Validation of DEmiRNAs

**Figure 7:**
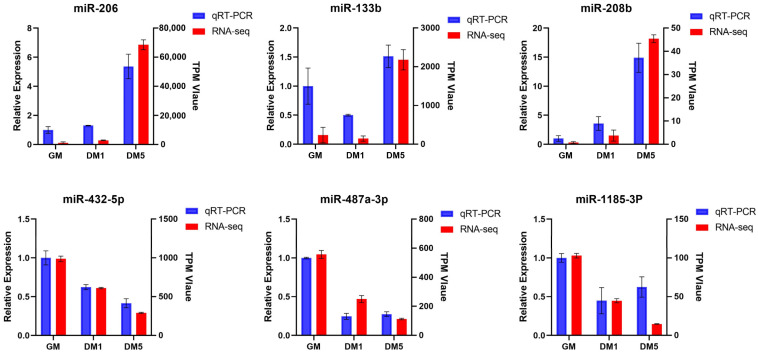
Validation of DEmiRNAs. Expression profiles of the six selected miRNAs based on RNA-Seq and qRT-PCR.

We validated the RNA-seq results through qRT-PCR analysis of selected DEmiRNAs. Specifically, we analyzed three miRNAs (miR-206, miR-133b, and miR-208b) that exhibited increased expression, as well as three miRNAs (miR-432-5p, miR-487a-3p, and miR-1185-3p) that demonstrated decreased expression (Figure 7). The qRT-PCR results correlated well with RNA-seq findings, thereby validating our sequencing data.

## 4. Discussion

Muscle mass and maturation are critical determinants of productivity and meat quality in livestock species, representing a central objective in animal breeding programs. MicroRNAs (miRNAs) are small regulatory RNAs (19–25 nucleotides) that modulate gene expression through translational inhibition and mRNA decay [13]. Advances in high-throughput sequencing technology and bioinformatics have recently enabled the identification of numerous miRNAs in livestock. These miRNAs regulate multiple aspects of muscle biology, encompassing stem cell activation, specification, migration, quiescence, myoblast proliferation and differentiation, and metabolic processes, ultimately influencing muscle formation [14,15,16]. Liao et al. [17] demonstrated that miR-99b-3p inhibits the intrinsic apoptosis pathway by interacting with caspase-3 and NCOR1, thereby promoting cell proliferation and preventing apoptosis in skeletal muscle satellite cells of Anhui white goats. Yun et al. [18] profiled biceps femoris from Wuranke sheep across three time points, revealing elevated oar-miR-133, bta-miR-1, and chi-miR-378-3p levels in postnatal animals (3 and 15 months) compared to fetuses. Conversely, chi-miR-206 and oar-miR-127 exhibited inverse expression dynamics, suggesting stage-specific regulatory roles for these miRNAs during ovine muscle development.

We performed miRNA-Seq on goat MuSCs under proliferative (GM) or differentiation conditions (DM1, DM5) to elucidate miRNA regulatory mechanisms in muscle formation. In this study, we identified 1846 miRNAs, with 677 differentially expressed across three comparison groups: 479 previously annotated and 198 newly discovered miRNAs. GO analysis demonstrated that DEmiRNA targets participate primarily in cellular proliferation, developmental regulation, and morphogenesis, processes essential for myogenesis. KEGG pathway analysis revealed several signaling cascades intricately linked to skeletal muscle development, including the Hippo pathway, cytokine–cytokine receptor interaction, TGF-β signaling pathway, and cell adhesion molecules (CAMs). These enrichments suggest that miRNA-mediated regulation may orchestrate myogenesis through stage-specific modulation of key signaling networks. Skeletal muscle maturation is a meticulously coordinated, sequential biological process, marked by myoblast cell cycle exit, differentiation into myocytes, and eventual fusion into multinucleated myotubes. In this context, we conducted clustering analysis on the dynamic expression profiles of DEmiRNAs across the three stages. One notable expression pattern (profile 0), comprising 171 miRNAs, exhibited sustained downregulation from the proliferative phase to the late differentiation stage. This pattern indicates that these DEmiRNAs may function as negative regulators during early myogenic differentiation, and their gradual downregulation likely facilitates the orderly progression of myoblast proliferation, differentiation, and fusion. Simultaneously, GO enrichment analysis revealed a significant enrichment of the target genes in biological processes such as development, cell differentiation, and cell proliferation. This observation aligns closely with the myogenic differentiation cascade, which includes myoblast cell cycle exit, differentiation commitment, and the ultimate formation of multinucleated, functionally specialized myotubes. Correspondingly, subsequent KEGG enrichment analysis corroborated that these target genes are predominantly involved in canonical myogenesis-related signaling pathways, including the Hippo, TGF-β, Hedgehog, and MAPK pathways.

Hippo signaling pathway genes (TEAD4, FGF1, PPP2R2C, and BIRC5) are closely related to goat meat production traits including carcass weight, some meat quality traits and muscle components [19]. The TGF-β signaling pathway is a crucial regulator in skeletal muscle development and regeneration, controlling myoblast fusion to affect myofiber nuclear content and fiber hypertrophy [20]. Shen et al. [21] demonstrated that miR-181-5p binds TGF-β receptor 1 (TGFBR1), suppressing SMAD2/3 phosphorylation in downstream signaling. The cytokine–cytokine receptor interaction pathway consists of cytokines and their receptors that mediate intercellular communication and signaling [22]. Many studies have confirmed the vital roles of key regulatory factors such as interleukin-6, myostatin, insulin-like growth factor 1, and tumor necrosis factor alpha in muscle formation [23,24,25,26]. Overall, miRNAs are critical regulators in goat skeletal muscle development through these relevant signaling pathways. Additionally, qRT-PCR validation of six randomly selected DEmiRNAs confirmed the reliability of the RNA sequencing data.

To further explore candidate miRNAs involved in goat skeletal MuSC development, we performed an integrative analysis and built a network of DEmiRNAs and mRNAs across all three developmental stages. We suggest that the newly identified miRNAs may affect muscle growth and development by targeting specific genes. In the interaction network, novel-m0047-5p exhibited strong, significant negative correlations with multiple muscle development-related target genes, including FOSB, CPT1B and MYOZ2. As a key AP-1 transcription factor subunit, FOSB forms leucine zipper heterodimers with JUN to regulate cell proliferation and differentiation [27]. Studies in bovine MuSCs have demonstrated that siRNA-mediated knockdown of FOSB markedly inhibited the expression of myogenic differentiation markers (MYH2, MYH3, MYOG, and CKM) and impeded myotube formation, thereby impairing muscle development [28]. In agreement with this observation, transcriptome analyses in pigs showed that FOSB expression was significantly elevated in the muscle of fast-growing breeds. Specifically, Moravka pigs exhibited higher FOSB levels than Mangalitsa pigs, associated with their superior growth rate and carcass weight [29]. Similarly, hybrid pigs with enhanced growth performance displayed significant FOSB upregulation compared to the slower-growing Huainan indigenous pigs [30]. At the mechanistic level, Cao et al. [31] reported that in common carp skeletal muscle, miR-203a-mediated repression of FOSB modulates translation elongation and autophagy via the AMPK–EEF2K axis, thereby promoting myogenesis. Recent single-cell transcriptomic analysis in goats further supports the conserved function of FOSB in muscle development. FOSB acts as a critical hub within the shared regulatory network of goat muscle satellite cells and fibro-adipogenic progenitors, displaying comparable expression dynamics in both populations. Through modulation of shared signaling pathways such as TGF-β, WNT and MAPK, FOSB coordinates the balance between myogenic and adipogenic processes during development [32].

CPT1B, a carnitine palmitoyltransferase 1 isoform primarily expressed in skeletal muscle and heart, catalyzes the transfer of long-chain fatty acyl moieties from coenzyme A to carnitine. This reaction represents a critical control point for mitochondrial fatty acid uptake and subsequent β-oxidation [33]. Studies in bovine *longissimus dorsi* muscle showed that CPT1B was significantly upregulated in the high-IMF group, and its expression was strongly positively correlated with IMF content. As a key candidate gene for IMF deposition, CPT1B participates in the fatty acid β-oxidation pathway, thereby modulating intramuscular fat accumulation [34]. Similarly, CPT1B expression was significantly increased in the *longissimus dorsi* of Songlei hybrid pigs compared with Songliao black pigs, consistent with their higher IMF content and backfat thickness [35].

MYOZ2 is a key component of the Z-disc in myofiber sarcomeres and plays an important regulatory role in muscle development and myofiber type determination [36]. siRNA-mediated knockdown of MYOZ2 in bovine *longissimus dorsi* muscle severely impaired myoblast differentiation and myotube formation, and significantly reduced the mRNA expression of key myogenic regulatory factors (MyoD, MyoG, MEF2A, and MyH). These results indicate that MYOZ2 functions in both the early commitment and late differentiation stages of myogenesis [37]. A study in Big-bone chickens showed that MYOZ2 expression was significantly higher in leg muscle than in breast muscle, supporting its role as a candidate differentially expressed gene associated with meat quality [38]. Furthermore, MYOZ2 knockout mice exhibited abnormal activation of the calcineurin signaling pathway in skeletal muscle. The absence of calsarcin-1 (the protein encoded by MYOZ2), a major negative regulator of this pathway, led to increased calcineurin activity. This in turn promoted the shift to slow-twitch myofiber specification via the CaN-NFAT axis, resulting in a marked increase in slow-twitch fiber proportion [39]. Accordingly, we hypothesize that novel-m0047-5p may exert regulatory effects by targeting FOSB, CPT1B, and MYOZ2, which in turn are potentially implicated in multiple signaling cascades including AMPK, TGF-β, fatty acid β-oxidation, and the CaN-NFAT pathway. This integrated regulation ultimately promotes myogenic differentiation, optimizes myofiber type composition, and enhances intramuscular fat deposition.

The miRNA-mRNA regulatory network elucidated in this study could serve as a foundational basis for the advancement of molecular breeding tools in meat goats. The candidate miRNAs, such as novel-m0047-5p, along with their target genes FOSB, CPT1B, and MYOZ2, emerge as potential molecular markers. Upon validation through functional studies at both cellular and organismal levels, these markers could be integrated into marker-assisted selection programs. Such integration may facilitate the early identification of superior breeding stock exhibiting enhanced muscle growth and improved meat quality traits, thereby potentially augmenting production efficiency and economic returns for farmers.

## 5. Conclusions

In this study, we characterized the miRNA expression dynamics during the proliferation and differentiation of goat skeletal muscle satellite cells. Functional enrichment analysis revealed that differentially expressed miRNAs were involved in TGF-β/Hippo signaling pathways and myogenic processes. Through integrated analysis of miRNA and transcriptome data, we constructed a regulatory network and identified key candidate miRNAs, including novel-m0047-5p, providing potential molecular targets for genetic improvement of goat meat quality traits. Nevertheless, the lack of experimental validation for these predicted miRNA–mRNA interactions represents a limitation of the present study.

## Figures and Tables

**Table 1:** Primer sequences for qRT-PCR validation.

miRNA	Primer Sequence (5′–3′)
miR-206	RT:GTCGTATCCAGTGCAGGGTCCGAGGTATTCGCACTGGATACGACACCACA
	F: CGACGCGATGGAATGTAAGGAAG
	R:ATCCAGTGCAGGGTCCGAGG
miR-133b	RT:GTCGTATCCAGTGCAGGGTCCGAGGTATTCGCACTGGATACGACAGCTGG
	F: GCGTTTGGTCCCCTTCAA
	R: AGTGCAGGGTCCGAGGTATT
miR-208b	RT:GTCGTATCCAGTGCAGGGTCCGAGGTATTCGCACTGGATACGACCAAACC
	F: GCGCGTATAAGACGAACAAAA
	R: AGTGCAGGGTCCGAGGTATT
miR-432-5p	RT:GTCGTATCCAGTGCAGGGTCCGAGGTATTCGCACTGGATACGACCCACCC
	F:CGCGTCTTGGAGTAGGTCATT
	R: AGTGCAGGGTCCGAGGTATT
miR-487a-3p	RT:GTCGTATCCAGTGCAGGGTCCGAGGTATTCGCACTGGATACGACAACTGG
	F: CGCGAATCATACAGGGACAT
	R: AGTGCAGGGTCCGAGGTATT
miR-1185-3p	RT:GTCGTATCCAGTGCAGGGTCCGAGGTATTCGCACTGGATACGACATAAGA
	F: AGCCAGCGATATACAGAGGGAGA
	R: ATCCAGTGCAGGGTCCGAGG
U6	F: CTCGCTTCGGCAGCACA
	R: AACGCTTCACGAATTTGCGT

**Table 2:** Summary of RNA-seq data.

Sample	Clean Reads (%)	High Quality (%)	3′Adapter Null (%)	Insert Null (%)	5′Adapter Contaminants (%)	polyA (%)	Clean Tags (%)
GM-1	12,481,378 (100%)	12,405,673 (99.3935%)	28,910 (0.2330%)	60,243 (0.4856%)	16,188 (0.1305%)	1272 (0.0103%)	12,299,060 (99.1406%)
GM-2	10,710,406 (100%)	10,653,299 (99.4668%)	21,606 (0.2028%)	32,514 (0.3052%)	15,896 (0.1492%)	796 (0.0075%)	10,582,487 (99.3353%)
GM-3	12,534,703 (100%)	12,439,528 (99.2407%)	17,799 (0.1431%)	60,081 (0.4830%)	13,355 (0.1074%)	937 (0.0075%)	12,347,356 (99.2590%)
DM1-1	14,858,148 (100%)	14,696,981 (98.9153%)	24,982 (0.1700%)	79,091 (0.5381%)	14,997 (0.1020%)	454 (0.0031%)	14,577,457 (99.1867%)
DM1-2	15,462,271 (100%)	15,342,202 (99.2235%)	32,126 (0.2094%)	56,054 (0.3654%)	13,736 (0.0895%)	414 (0.0027%)	15,239,872 (99.3330%)
DM1-3	17,300,792 (100%)	17,203,942 (99.4402%)	38,487 (0.2237%)	56,474 (0.3283%)	13,800 (0.0802%)	278 (0.0016%)	17,094,903 (99.3662%)
DM5-1	15,513,942 (100%)	15,415,116 (99.3630%)	33,078 (0.2146%)	40,804 (0.2647%)	13,045 (0.0846%)	460 (0.0030%)	15,327,729 (99.4331%)
DM5-2	11,596,025 (100%)	11,533,653 (99.4621%)	23,084 (0.2001%)	33,276 (0.2885%)	8501 (0.0737%)	325 (0.0028%)	11,468,467 (99.4348%)
DM5-3	12,902,085 (100%)	12,789,716 (99.1291%)	30,950 (0.2420%)	27,689 (0.2165%)	8495 (0.0664%)	325 (0.0025%)	12,722,257 (99.4726%)

## Data Availability

The RNA-seq data for goat skeletal muscle satellite cells (MuSCs) have been deposited in the NCBI with the primary accession code PRJNA779184.

## Supplementary Materials

The following supporting information can be downloaded at: https://www.mdpi.com/article/10.3390/cells15060519/s1, Table S1: Distribution of high-quality read lengths; Table S2: Count of DEmiRNAs in GM vs. DM1, GM vs. DM5, and DM1 vs. DM5; Table S3: Table of DEmiRNAs and target genes in the GM vs. DM1, GM vs. DM5, and DM1 vs. DM5 group results; Table S4: Significantly enriched GO enrichment analysis for GM vs. DM1; Table S5: Significantly enriched GO enrichment analysis for GM vs. DM5; Table S6: Significantly enriched GO enrichment analysis for DM1 vs. DM5; Table S7: Significant enrichment of KEGG pathways by DEmiRNAs in the GM vs. DM1, GM vs. DM5, and DM1 vs. DM5 groups; Table S8: DEmiRNAs-DEmRNAs co-expression network; Table S9: DEmiRNAs-DEmRNAs correlation analysis.
